# Copolymer-Coated Gold Nanoparticles: Enhanced Stability and Customizable Functionalization for Biological Assays

**DOI:** 10.3390/bios14070319

**Published:** 2024-06-24

**Authors:** Dario Brambilla, Federica Panico, Lorenzo Zarini, Alessandro Mussida, Anna M. Ferretti, Mete Aslan, M. Selim Ünlü, Marcella Chiari

**Affiliations:** 1 Institute of Chemical and Technological Science “Giulio Natta”, National Research Council of Italy, Via Privata Mario Bianco 9, 20131 Milan, Italy; federica.panico@scitec.cnr.it (F.P.); lorenzo.zarini@scitec.cnr.it (L.Z.); alessandro.mussida@scitec.cnr.it (A.M.); marcella.chiari@scitec.cnr.it (M.C.); 2 Institute of Chemical and Technological Science “Giulio Natta”, National Research Council of Italy, Via Gaudenzio Fantoli 16/15, 20138 Milan, Italy; anna.ferretti@scitec.cnr.it; 3 Electrical and Computer Engineering Department, Boston University, Boston, MA 02215, USA; maslan@bu.edu (M.A.); selim@bu.edu (M.S.Ü.)

**Keywords:** gold nanoparticles, coating, polymer, biomolecule functionalization, bioassay, microarray, streptavidin, DNA

## Abstract

Gold nanoparticles (AuNPs) play a vital role in biotechnology, medicine, and diagnostics due to their unique optical properties. Their conjugation with antibodies, antigens, proteins, or nucleic acids enables precise targeting and enhances biosensing capabilities. Functionalized AuNPs, however, may experience reduced stability, leading to aggregation or loss of functionality, especially in complex biological environments. Additionally, they can show non-specific binding to unintended targets, impairing assay specificity. Within this work, citrate-stabilized and silica-coated AuNPs (GNPs and SiGNPs, respectively) have been coated using *N*,*N*-dimethylacrylamide-based copolymers to increase their stability and enable their functionalization with biomolecules. AuNP stability after modification has been assessed by a combination of techniques including spectrophotometric characterization, nanoparticle tracking analysis, transmission electron microscopy and functional microarray tests. Two different copolymers were identified to provide a stable coating of AuNPs while enabling further modification through click chemistry reactions, due to the presence of azide groups in the polymers. Following this experimental design, AuNPs decorated with ssDNA and streptavidin were synthesized and successfully used in a biological assay. In conclusion, a functionalization scheme for AuNPs has been developed that offers ease of modification, often requiring single steps and short incubation time. The obtained functionalized AuNPs offer considerable flexibility, as the functionalization protocol can be personalized to match requirements of multiple assays.

## 1. Introduction

Gold nanoparticles (AuNPs) conjugated to biomolecules are crucial for various applications in biotechnology, medicine, and diagnostics [1,2,3]. Despite being derived from a material known for its costliness, AuNPs are economically viable due to their stability over extended periods and their effective utilization at low concentrations. The distinctive optical properties of gold nanoparticles, including their strong surface plasmon resonance and intense scattering and absorption properties, make them useful as contrast agents for various imaging techniques, including electron microscopy, optical microscopy, and lateral flow assays [4,5,6]. The conjugation with antibodies, antigens, proteins, or nucleic acids enables the binding of the AuNPs to desired biomolecular targets minimizing interference from other components present in the sample. When conjugated with specific biological molecules, AuNPs provide high sensitivity and multiplexing capabilities in biosensing, facilitating the detection of various targets through colorimetric, fluorescent, or surface-enhanced Raman scattering (SERS) detection methods [7,8]. AuNP-based sensor platforms with microfluidic devices result in the development of portable, point-of-care diagnostic tools applicable across healthcare, food safety, and environmental monitoring sectors [9]. The necessary stage in incorporating AuNPs into any sensing platform requires their modification with biomolecular ligands. However, the process of conjugation presents numerous obstacles. Preserving the functionality of biomolecules during conjugation is paramount to maintain assay performance and accuracy. The functionalization of AuNPs with large biomolecules or multiple ligands may introduce steric hindrance effects, which could impact the accessibility of binding sites or the efficiency of target recognition. Therefore, optimizing the spacing and orientation of biomolecules on AuNPs is crucial for minimizing steric hindrance effects and maximizing assay sensitivity [10]. Additionally, functionalized AuNPs may exhibit reduced stability compared to bare nanoparticles, especially in complex biological environments or harsh experimental conditions. Instability can lead to aggregation, degradation, or loss of functionality over time, affecting the reliability and reproducibility of experimental results [11]. This aggregation process can occur due to electrostatic interactions. When functionalized with charged ligands or molecules, AuNPs may experience electrostatic repulsion or attraction depending on the charge of the functional groups [12]. Another problem is non-specific binding to unintended targets or surfaces, leading to background signals and reduced assay specificity. Minimizing non-specific binding is crucial for improving assay sensitivity and accuracy, particularly in complex biological samples [13].

This article presents a novel method for surface modification and functionalization of AuNPs, which addresses several of the challenges outlined above. The approach involves chemisorption of polymers containing click chemistry functional groups, effectively resolving many of the issues encountered in conventional methods. Copolymers derived from *N*,*N*-dimethylacrylamide (copoly-DMA) have emerged as highly versatile coatings suitable for a wide range of applications [14]. Notably, they have found extensive use in the development of microarrays and multispot biosensors on various substrates, as supported by relevant references [15]. This class of copolymers offers rapid and durable surface adhesion, facilitating the covalent attachment of biomolecules while exhibiting excellent antifouling properties [16]. The key precursor of these copolymers, *N*-acryloyloxysuccinimide (NAS), features a functional moiety that readily reacts with functional groups conducive to click-chemistry reactions, such as azide/alkyne reactions [14]. Furthermore, click-chemistry reactions facilitate the biorthogonal orientation of immobilized probes.

Since the polymer is known to form a nanometric film on the surface of silica, we have utilized AuNPs encapsulated within a silica shell through a sol-gel process. The silica shell provides stability, biocompatibility, and a versatile platform for further functionalization with a copolymer of dimethylacrylamide. This represents a significant advancement with regard to available methods to produce a stable polymeric coating on gold nanoparticles. These methods usually require long incubation times to graft the polymer on the nanoparticle [17,18] or employ cumbersome protocols to grow the polymer directly on initiator-functionalized AuNPs [19,20,21]. The method herein described provides a stable coating in shorter times and in a greener way since it only requires water as a buffer.

In this work, we demonstrate that dimethylacrylamide polymers can also be used to coat AuNPs lacking a silica shell. Uncoated gold nanoparticles present a unique opportunity for modification with thiol-bearing reagents. However, the inherent challenge lies in the limited colloidal stability of AuNPs during the conjugation process, which may lead to aggregation. Aggregation propensity was observed also during the polymer coating stage. Indeed, the polymers employed in this investigation offer a significant advantage, thanks to their exceptional versatility. This adaptability allows for modifications to their composition, either during synthesis or through post-polymerization processes. To coat AuNPs without inducing aggregation, we introduced an ionizable monomer into the polymer backbone. This strategic modification not only ensures the stable coating of AuNPs but also creates opportunities for diverse functionalization, thereby enhancing their applicability across various fields. The polymer film bearing PEG-azide functionalities allows covalent binding of proteins and DNA modified with dibenzocyclooctyne (DBCO), a group characterized by its high reactivity towards azides via a copper-free strain-promoted alkyne-azide cycloaddition (SPAAC) reaction [22]. This reaction proceeds rapidly and efficiently without the need for a copper catalyst, making it particularly attractive for bioconjugation and labeling applications in biological systems where copper could be cytotoxic. 

We showcase the utility of DNA and streptavidin-conjugated nanoparticles in the Single Particle Interferometric Reflectance Imaging Sensor (SP-IRIS) [23]. This innovative technique combines the principles of interferometry and microscopy to provide high-resolution imaging of individual nanoparticles tethered to a substrate. 

## 2. Materials and Methods

### 2.1. Materials

Ammonium sulfate ((NH_4_)_2_SO_4_), phosphate buffer saline tablets (PBS), Tween20, sucrose monolaurate, sodium phosphate (Na_3_PO_4_), sodium chloride (NaCl), ethanolamine, trehalose dihydrate, magnesium chloride (MgCl_2_), sodium azide (NaN_3_), saline-sodium citrate buffer (SSC), *N*,*N*-Dimethylacrylamide, *N*-acryloyl succinimide, 3-(Trimethoxysilyl)propyl methacrylate, Immobiline Buffers pKa = 10.3, 11-azido-3,6,9-trioxaundecan-1-amine, tetrahydrofuran (THF), petroleum ether, 2,2′-azobis(2-metilpropionitrile) (AIBN) streptavidin, dibenzocyclooctyne-*N*-hydroxysuccinimide ester (DBCO-NHS ester), and Amicon Ultra 10 MWCO centrifugal filters were purchased from Sigma Aldrich (St. Louis, MO, USA). Oligonucleotides were synthesized by MWG-Biotech AG (Ebevsberg, Germany). Oligonucleotides were freeze-dried and resuspended in deionized water (DI water) at a final concentration of 100 µM before use. Untreated silicon chips with 110 nm thermal grown oxide were supplied by IRISkinetics (Boston, MA, USA). Chips were pretreated using a HARRICK Plasma Cleaner, PDC-002 (Ithaca, NY, USA), connected to an oxygen line. Copoly azide 4% copolymer (4% of azide groups) was synthesized as reported elsewhere [14]. MCP-4 is the trade name of a polymer purchased from Lucidant Polymers Inc. (Sunnyvale, CA, USA). Silica-coated gold nanoparticles with an outer silica shell of 3 nm were purchased from CD Bioparticles (Shirley, NY, USA). Gold nanoparticles of 40 nm in diameter, citrate-stabilized and supplied in water, were purchased from DCN (Carlsbad, CA, USA). 

### 2.2. Instrumentation

Nanoparticle Tracking Analysis was performed with NanoSight NS300 using 3.2 Dev Build 3.2.16 software (Malvern Instruments Ltd., Malvern, UK). Antibody Aptamer Conjugates were purified using proFIRE instrument (Dynamic Biosensors GmbH, Munchen, Germany). Spectrophotometric characterizations were performed with Multiskan SkyHigh instrument from Thermo Scientific. Samples containing gold nanoparticles were sonicated using Omni Ruptor 250-Watt Ultrasonic Cell Disruptor (OMNI International, GA, USA). Images were created using Biorender (www.biorender.com). The Single Particle IRIS (SP-IRIS) images were acquired every 2 min by scanning 20 μm with 1 μm step size using a conventional SP-IRIS setup [23]. After every defocus stack was normalized by its median along the defocus dimension, the SP-IRIS signal was constructed by calculating the difference between the maximum and minimum value of every stack.

### 2.3. Oligonucleotide Sequences 

DNA sequences were modified as reported (amine and azide linkers were linked at the 5′ end, while DBCO and biotin were linked to the 3′ end or 5′ end). Stabilizer DNA sequence was used without modification. Table 1 lists the sequences used in this work.

### 2.4. Synthesis of DMA-Based Copolymers

#### 2.4.1. Synthesis of Copoly NAS positive 4%

The polymer was synthesized by free radical polymerization as reported in [14]. Briefly, in a two-neck round-bottom flask, 10 mL of anhydrous THF were degassed for 20 min by insufflating argon. After that, DMA (1.967 g, 19.85 mmol, 2.044 mL), NAS (0.142 g, 0.84 mmol), MAPS (0.052 g, 0.21 mmol, 0.050 mL) and immobiline buffer pKa 10.3 (0.525 mL of 200 mM solution in isopropanol, 0.105 mmol) were added under argon atmosphere. The immobiline buffer was dried under reduced pressure and all the monomers were suspended in 0,5 mL of anhydrous THF. To the resulting reaction mixture was then added AIBN (5 mg) under argon atmosphere and the polymerization was conducted at 65 °C for 2 h.

The reaction mixture was cooled to room temperature and 10 mL of anhydrous THF were added (polymer concentration 10% *w*/*v*). The polymer was then precipitated in 200 mL of petroleum ether and left stirring for 1.5 h. The polymer was then filtered on a Buchner and dried under vacuum at room temperature.

#### 2.4.2. Synthesis of Copoly Azide Positive 4%

The polymer was synthesized by post-polymerization modification of copoly NAS positive 4%, similarly to what was described in [14]. In a two-neck round-bottom flask, copoly NAS positive 4% (0.5 g, 0.0483 mmol) was solubilized with 5 mL of anhydrous THF and degassed for 5 min by insufflating argon. 11-azido-3,6,9-trioxaundecan-1-amine (0.105 g, 0.483 mmol) was then added to the reaction mixture and the reaction proceeded at room temperature for 5 h.

The reaction mixture was cooled to room temperature. The polymer was then precipitated in 50 mL of petroleum ether and left stirring for 1.5 h. The polymer was then filtered on a Buchner and dried under vacuum at room temperature.

### 2.5. Functionalization of Microarray Chips (General Procedure)

60 nm silicon oxide SP-IRIS supports were pretreated with oxygen plasma to clean and activate the surface. The oxygen pressure was set to 1.2 bar with a power of 29.6 W for 10 min. Then chips were dipped into a 1% *w*/*v* aqueous solution of MCP-4 in 0.45 M ammonium sulfate. The supports were immersed into the coating solution for 30 min at room temperature, rinsed with bidistilled water, dried under nitrogen stream, and finally cured at 80 °C for 15 min.

Supports were spotted using a noncontact microarray spotter (sciFLEXARRAYER S12, Scienion, Berlin) equipped with an 80 µm nozzle. 400 pL of solution were printed at room temperature and 65% humidity. 

To prepare spotting solutions, oligonucleotides were diluted in a solution of 150 mM sodium phosphate buffer containing 0.01% sucrose monolaurate at pH 8.5. After spotting, chips were stored overnight in a sealed chamber filled at the bottom with a sodium chloride saturated solution (40 g/100 mL, 65% humidity). Finally, chips were treated with a blocking solution containing ethanolamine (50 mM in 0.1 M Tris/HCl buffer pH 9 and 2 mM MgCl_2_) at room temperature for 1 h, rinsed with MgCl_2_ 2 mM, and dried.

### 2.6. Nanoparticle Tracking Analysis 

GNPs and silica-coated GNPs were analyzed using Nanosight NS300 (Malvern Panalytical, Malvern, UK). Videos were analyzed by the in-built NanoSight Software NTA 3.4 Dev Build 3.2.16. The Camera type, Camera level, and Detect Threshold were sCMOS, 9 and 5, respectively, for 80 nm nanoparticles and 11 and 5, respectively for 40 nm nanoparticles. The number of completed tracks in NTA measurements was 5 (a 60-s movie was registered for each measurement). Samples were diluted in MQ water to a final volume of 1 mL. The ideal particle concentration was assessed by pre-testing the optimal particle per frame value (20-100 particles per frame).

### 2.7. Synthesis of DBCO-Modified Streptavidin

To 300 µL of 2 mg/mL streptavidin in PBS, 5.4 µL of 4 mM DBCO-NHS ester were added. The mixture was incubated for 30 min at 25 °C. After the incubation, 30 μL of Tris-HCl 1 M pH 8 were added, and the reaction was allowed to proceed for 5 min. The DBCO-modified streptavidin was then purified using Amicon Ultra 10 MWCO centrifugal filters (3 × 5 min at 12,200× *g*) and finally PBS was added to bring the volume to 300 µL.

### 2.8. Coating of Silica-Coated GNPs (SiGNPs) with Copoly Azide 4%

Coating and functionalization procedures were adapted from [16]. Silica-coated gold nanoparticles were vortexed for 30 s and then sonicated using a bath sonicator for 10 min. Subsequently, a solution of Copoly Azide 4% at 1% *w*/*v* was prepared. SiGNPs were diluted to a final concentration of 1.4 OD by suspending 20 µL of SiGNPs in 480 µL of the polymer solution; the sample was incubated for 1 h at 25 °C under stirring. At the end of the incubation, the sample was centrifuged for 5 min at 12,200× *g*, the supernatant was removed, and SiGNPs resuspended in 500 µL of MQ water. Centrifugation was repeated three times to wash SiGNPs. Finally, SiGNPs were redispersed using an immersion sonicator. The same protocol was used for both 40 nm and 80 nm silica-coated GNPs.

### 2.9. Coating of GNPs with Copoly Azide Positive 4%

40 nm GNPs were vortexed for 30 s and then sonicated using a bath sonicator for 10 min. Subsequently, a 2% *w*/*v* solution of copoly azide positive 4% was prepared. GNPs were diluted to a final concentration of 0.6 OD by suspending 250 µL of GNPs in 250 µL of a 2% *w*/*v* solution of copoly azide positive 4% in water; the sample was incubated for 30 min at 25 °C under stirring. At the end of the incubation, the sample was centrifuged for 2 min at 6720× *g*, the supernatant was removed, and GNPs resuspended in 250 µL of MQ water. Centrifugation was repeated three times to wash GNPs. Finally, GNPs were redispersed in 250 µL of MQ water using an immersion sonicator.

### 2.10. Functionalization of Coated SiGNPs with ssDNA

200 μL of copoly azide 4% coated 80 nm SiGNPs (prepared as described in Section 2.8) were centrifuged for 5 min at 12,000× *g*, the supernatant was removed, and SiGNPs were resuspended in 200 μL of 10 μM DBCO-modified ssDNA in PBS and incubated overnight at 37 °C under stirring. At the end of the incubation, the sample was centrifuged for 5 min at 12,000× *g*, the supernatant was removed, and SiGNPs resuspended in 200 μL of MQ water. Centrifugation was repeated three times to wash GNPs. Finally, SiGNPs were redispersed using an immersion sonicator.

### 2.11. Functionalization of Copoly azide 4% Coated SiGNPs with Streptavidin

To a 200 µL solution of 80 nm SiGNPs coated with copoly azide 4% (prepared as described in Section 2.8), 30 µL of 2 mg/mL DBCO-modified streptavidin, 70 µL of MQ water, and 0.2 µL of Tween 20 were added and the obtained solution was incubated overnight at 25 °C under stirring. After the incubation, the sample was centrifuged for 5 min at 12,000× *g*, the supernatant was removed, and SiGNPs resuspended in 300 µL of 0.1X PBS + 0.05% Tween 20. Centrifugation was repeated three times to wash SiGNPs. Finally, SiGNPs were redispersed using an immersion sonicator.

### 2.12. Functionalization of Copoly Azide Positive 4% Coated GNPs with Streptavidin

To a 200 µL solution of 40 nm GNPs coated with copoly azide positive 4% (prepared as described in Section 2.9), 30 µL of 2 mg/mL DBCO-modified streptavidin, 70 µL of MQ water, and 0.2 µL of Tween 20 were added and the obtained solution was incubated overnight at 25 °C under stirring. After the incubation, the sample was centrifuged for 2 min at 10,000× *g*, the supernatant was removed, and GNPs resuspended in 300 µL of 0.1X PBS + 0.05% Tween 20. Centrifugation was repeated three times to wash GNPs. Finally, GNPs were redispersed using an immersion sonicator.

### 2.13. Spectrophotometric Characterization

200 μL of each sample of GNPs suspension were placed in individual wells in a 96-well plate. Spectra between 400 and 1000 nm were acquired at room temperature.

### 2.14. Transmission Electron Microscopy

The TEM images were recorded by ZEISS Libra 200 FE 200kV equipped with Omega filter in column. The samples were prepared by dropping the suspension on copper TEM grid and letting it dry in air. The diameter measurements were performed by the iTEM TEM Imaging Platform software (TEM Server 7.5; Olympus, Miami, FL, USA).

### 2.15. Stability Test

200 μL of 40 nm SiGNPs (both uncoated and coated with copoly azide 4%) and GNPs (both uncoated and coated with copoly azide positive 4%) were suspended in a solution of HCl 0.1 M or NaOH 0.1 M. A simple visual test was performed to evaluate the aggregation and precipitation state of nanoparticles and absorbance spectra were acquired as described in Section 2.13.

### 2.16. Functional Test using ssDNA-Functionalized SiGNPs

SP-IRIS chips were functionalized with Probe2 and Probe7 (the negative and the positive spots, respectively) as described in Section 2.5. Chips were mounted on the support slide and positioned inside the SP-IRIS instrument. Chips were washed by flushing twice with 500 µL of MQ water and once with 500 µL of 2X SSC (each washing step was performed at 500 µL/min). Chips were incubated with a solution of 10 nM Utag-Tag7 in 2X SSC for 40 min at 10 μL/min. At the end of the first incubation, chips were washed by flushing 2X SSC and then incubated with 0.1 OD of Utag-functionalized SiGNPs (prepared as described in Section 2.10) in 2X SSC for 40 min at 10 μL/min. During the incubation, a single image was acquired every 2 min. Then, acquired images were processed and SiGNPs immobilized on spots were counted using ImageJ software (ImageJ 2.16).

### 2.17. Functional Test Using Streptavidin-Functionalized SiGNPs

SP-IRIS chips were functionalized with Probe2 and Probe7 (the negative and the positive spots, respectively) as described in Section 2.5. Chips were mounted on the support slide and positioned inside the SP-IRIS instrument. The chip was washed by flushing twice with 500 µL of MQ water and once with 500 µL of 2X SSC (each washing step was performed at 500 µL/min). The chip was incubated with 10 nM Utag-Tag7 in 2X SSC for 40 min at 10 μL/min. At the end of the incubation, the chip was washed by flushing 2X SSC and then incubated with 100 nM Utag-Biotin in 2X SSC for 40 min at 10 µL/min. At the end of the incubation, the chip was washed by flushing 2X SSC and then incubated with 0.07 OD solution of streptavidin-functionalized SiGNPs in 2X SSC for 40 min at 10 μL/min. During the last incubation, a single image was acquired every 2 min. Then, acquired images were processed and particles immobilized on spots were counted using ImageJ software.

## 3. Results

**Figure 1:**
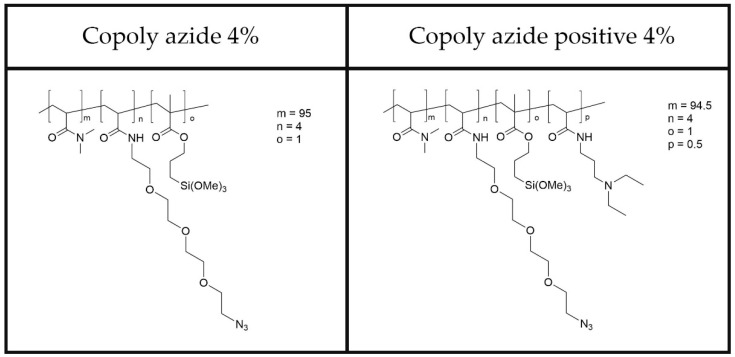
Chemical structures of copolymers used in this work.

In this work, we report the coating of two classes of nanoparticles: gold nanoparticles coated with a thin silica layer (SiGNPs) and citrate-stabilized AuNPs (GNPs). Both materials were functionalized through adsorption of dimethylacrylamide polymers bearing azido groups. The chemical structure of the two polymers is reported in Figure 1. These polymers are characterized by biocompatibility, ease of application on surfaces, and fouling-resistant abilities. The experimental conditions such as polymer concentration, incubation time, and temperature to achieve the desired coating thickness and stability were optimized.

To a water colloidal suspension of commercially available gold nanoparticles (either GNPs or SiGNPs), a 1% *w*/*v* aqueous solution of polymer was added under stirring. Water is compatible with both the polymer and the dispersion medium of the gold nanoparticles. Short incubation times (up to 1 h) allowed formation of a nanometer-thick polymer onto the gold nanoparticle surface. After coating, AuNPs were centrifuged and washed several times with water to eliminate any residual unbound polymer or impurities. 

### 3.1. Vis Spectroscopy 

A spectrophotometer equipped with a Vis light source and a detector was used to characterize the colloidal suspension of coated nanoparticles. When needed, the nanoparticles were dispersed to form a colloidal suspension free from aggregates by sonication. The absorption spectra were recorded by scanning the wavelength range typically from 400 nm to 1000 nm, covering the infrared and visible regions. 

**Scheme 1:**
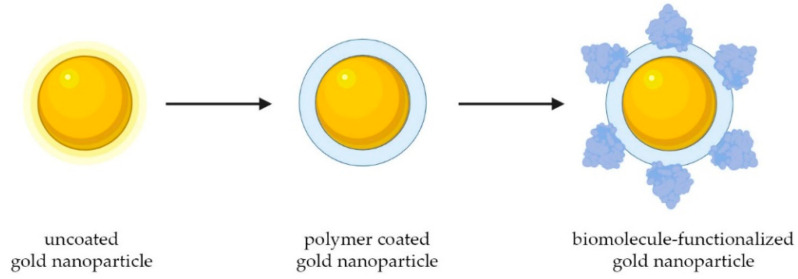
Schematic representation of the functionalization pathway followed to modify AuNPs.

As shown in Scheme 1, we have bound oligonucleotides and streptavidin to the surface of both AuNPs, a ligand that allows grafting of different biotin modified biomacromolecules.

**Figure 2:**
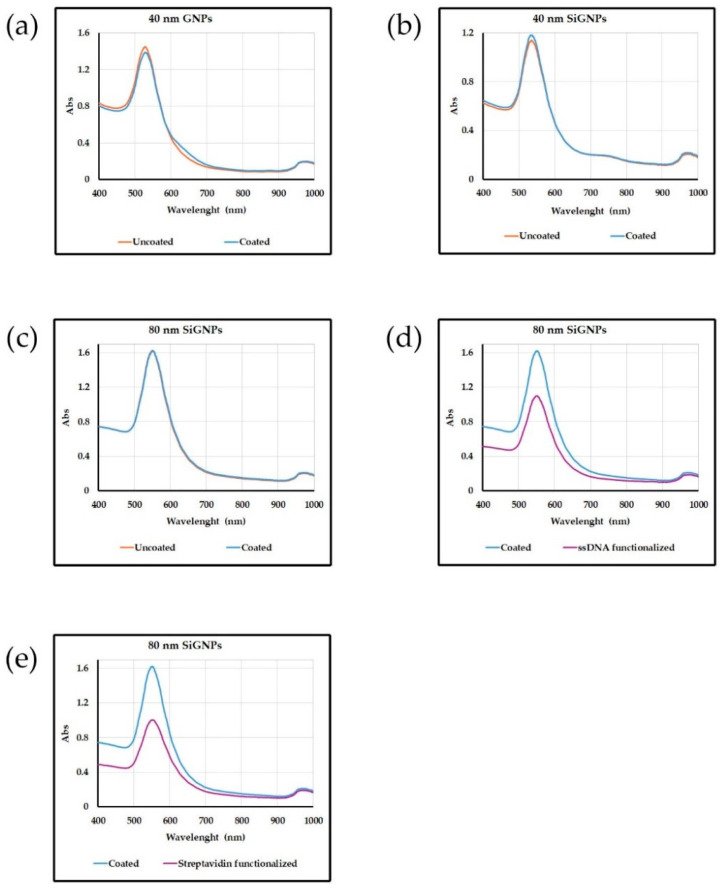
Absorption spectra of gold nanoparticles (both SiGNPs and GNPs) at various stages of functionalization: (**a**) 40 nm GNPs uncoated and coated with copoly azide positive 4%; (**b**) 40 nm SiGNPs uncoated and coated with copoly azide 4%; (**c**) 80 nm SiGNPs uncoated and coated with copoly azide 4%; (**d**) 80 nm SiGNPs functionalized with ssDNA; (**e**) 80 nm SiGNPs functionalized with streptavidin.

The absorption spectra shown in Figure 2 exhibit one distinct peak at a wavelength corresponding to the SPR of gold nanoparticles. The position, shape, and intensity of the peak confirm that the nanoparticles are not aggregated, and the diameter of the particles is 40 or 80 nm. The intensity of the peak is proportional to the concentration of nanoparticles in the sample, which is close to that of the uncoated starting beads indicating that the coating proceeds without loss of material. 

### 3.2. Nanoparticle Tracking Analysis (NTA)

**Figure 3:**
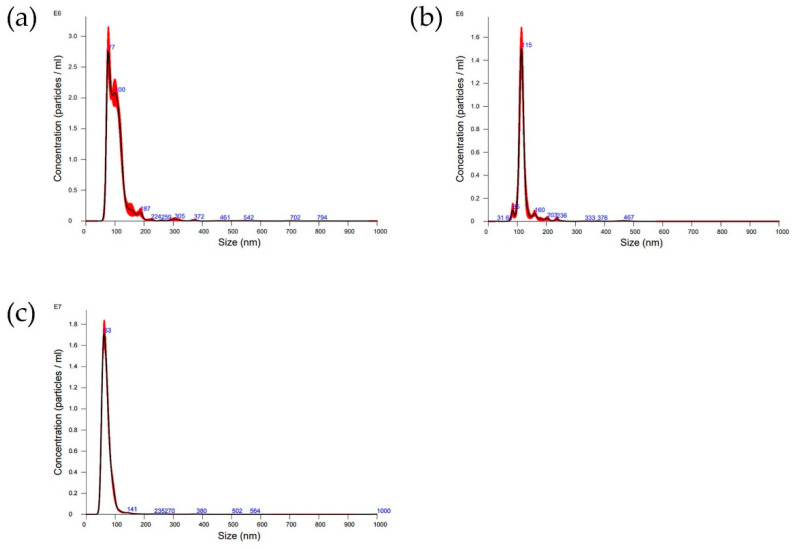
NTA analysis of: (**a**) 40 nm SiGNPs coated with copoly azide 4%; (**b**) 80 nm SiGNPs coated with copoly azide 4%; (**c**) 40 nm GNPs coated with copoly azide positive 4%.

Figure 3 illustrates the size distribution derived from NTA analysis of polymer-modified nanoparticles for particles nominally sized at (a,c) 40 and (b) 80 nm. Mean size values and standard deviations resulting from three measurements of each sample are reported in Table S1. All size values diverge from those stipulated by the manufacturer. This discrepancy may arise because NTA measures the hydrodynamic radius in solution, which could encompass ions surrounding the gold particles. In all instances, the introduction of a polymeric coating prompts an increase in particle size as discerned by NTA. This implies that the soft coating induces an expansion in the hydrodynamic radii caused by phenomena like swelling and solvation. Often, the further functionalization with biomolecules results in a reduction in the hydrodynamic radius. This might be due to the fact that binding with proteins and DNA reduces the capability of the polymer layer to retain water, thus causing a release in the size measured by NTA (see Table S1).

### 3.3. Transmission Electron Microscopy (TEM)

TEM is a powerful imaging technique used to visualize the internal structure and surface morphology of nanoparticles at a very high resolution. 

The morphological TEM characterization of the 40 nm SiGNPs samples, uncoated and ssDNA-functionalized, is reported in Figure S1. The SiGNPs shape is spherical at each functionalization step, and the mean diameter is 44.9 ± 2.8 nm, 44.4 ± 2.8 nm, and 44.8 ± 2.9 nm for uncoated, polymer-coated and ss-DNA-functionalized SiGNPs, respectively. The size distribution of each sample is highly monodispersed (see Figure S2). These findings confirm that the treatments do not affect the GNP morphology. 

### 3.4. Stability Tests

Stability tests of gold nanoparticles are crucial to understand their behavior in various environments and applications. We conducted a stability test by exposing the SiGNPs (both uncoated and coated with copoly azide 4%) and GNPs (both uncoated and coated with copoly azide positive 4%) to a solution of HCl 0.1 M or NaOH 0.1 M. Solution properties were analyzed by acquiring absorbance spectra between 400 and 1000 nm. Macroscopic changes can also be appreciated by the naked eye, since poor stability usually leads to aggregation. In a first step, small AuNPs aggregate to form nanometric entities, and this usually results in a color shift of the solution from red to blue. If the stability is poor, aggregation continues until microparticles are formed due to massive interactions between aggregates. In this case, the solution becomes colorless.

**Figure 4:**
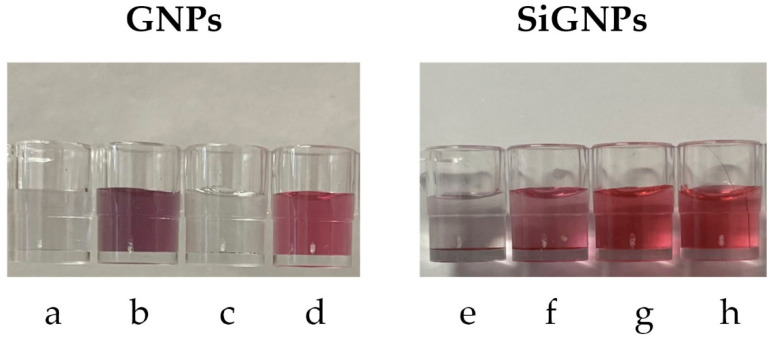
Stability test conducted on GNPs: (**a**) uncoated GNPs in 0.1 M HCl; (**b**) polymer-coated GNPs in 0.1 M HCl; (**c**) uncoated GNPs in 0.1 M NaOH; (**d**) polymer-coated GNPs in 0.1 M NaOH. Stability test conducted on SiGNPs: (**e**) uncoated SiGNPs in 0.1 M HCl; (**f**) polymer-coated SiGNPs in 0.1 M HCl; (**g**) uncoated SiGNPs in 0.1 M NaOH; (**h**) polymer coated SiGNPs in 0.1 M NaOH.

As regards citrate-stabilized GNPs, they show poor stability in both acidic and basic conditions, as evidenced by the loss of color (Figure 4a,c). The coating with copoly azide positive 4% sensibly reduces the aggregation in both conditions, especially the stability in NaOH, as shown in Figure 4b,d.

Considering SiGNPs, the silica shell provides superior stability in basic conditions, while acidic buffers still cause aggregation of nanoparticles (Figure 4e,g). Coating the SiGNPs with copoly azide 4% improves the stability in HCl while maintaining the stability in basic conditions (Figure 4f and Figure 4h, respectively).

**Figure 5:**
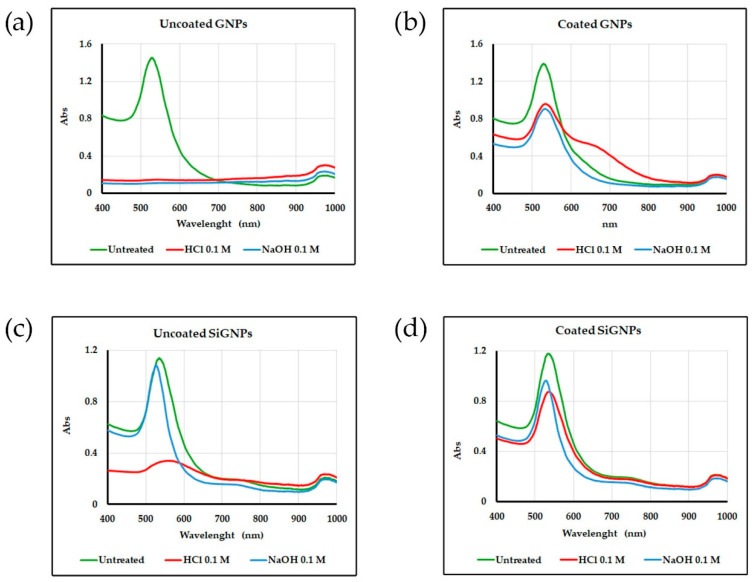
Vis spectra for untreated AuNPs and AuNPs treated with 0.1 M HCl or 0.1 M NaOH. (**a**) Spectra of uncoated GNPs; (**b**) Spectra of coated GNPs; (**c**) Spectra of uncoated SiGNPs; (**d**) Spectra of coated SiGNPs.

Absorbance spectra of the same solutions are reported in Figure 5. As can be noted, uncoated AuNPs show limited stability (highlighted by the disappearance of the SPR peak for GNPs and by the decrease in its height in SiGNPs samples). The stability improves when polymer coating is performed. A slight aggregation can be noted for GNPs treated with HCl, as suggested by the broad shoulder between 600 and 700 nm.

### 3.5. SiGNPs Functionalization with Biomolecules

In a plethora of biological assays, biomolecules (including DNA, proteins and peptides) are immobilized on AuNPs to provide labeling of analytes within the assay. The choice of the optimal modification strategy strongly influences the overall performance of the entire assay itself in terms of specificity, sensitivity, and nonspecific binding (thus impacting the signal-to-noise ratio and limit of detection). In this work, 80 nm SiGNPs were coated with copoly azide 4%. The polymer contains azide groups that are capable of reacting with DBCO-modified biomolecules. Conjugation occurred through incubating the polymer-coated SiGNPs with the biomolecule solution under appropriate conditions (pH, temperature, buffer), resulting in biomolecules binding to azide groups on the SiGNP surface via covalent bonding. 

**Figure 6:**
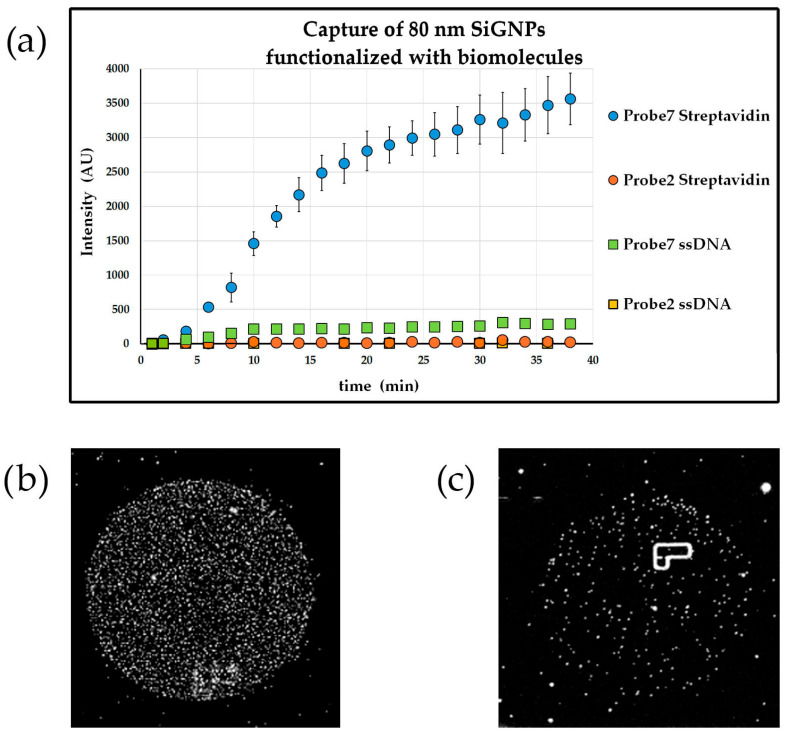
Single molecule detection experiment using SP-IRIS instrument. (**a**) Binding of 80 nm SiGNPs functionalized with ssDNA (squares) or streptavidin (circles); (**b**) image of a single spot of Probe7 after incubation with SiGNPs functionalized with streptavidin; (**c**) image of a single spot of Probe7 after incubation with SiGNPs functionalized with ssDNA. Every white dot in pictures (**b**) and (**c**) represents an individual SiGNP bound to the surface of the spot. The “L” shapes on the spots are reference regions patterned on the surface of the chip to find the locations of spots on the surface.

Following this experimental scheme, SiGNPs decorated with a ssDNA sequence (called Utag) or streptavidin were synthesized. Absorption spectra for functionalized SiGNPs are reported in Figure 2d,e. The performance of the so-obtained functionalized AuNPs was assessed using SP-IRIS prototype that is able to count individual nanoparticles bound to the surface of a microarray chip as described in Section 2.16 and Section 2.17. Briefly, chips were functionalized with two sequences of DNA, namely Probe2 (the negative control) and Probe7. The chips were incubated with Utag-Tag7 that binds to Probe7. Then, SiGNPs bind to Utag-Tag7 directly (in the case of Utag-functionalized SiGNPs) or through Utag-Biotin sequence (when streptavidin-functionalized SiGNPs are used). Results (see Figure 6) show that both SiGNPs (functionalized with either Utag or streptavidin) can bind selectively to positive spots (i.e., Probe7) with low nonspecific signals on negative spots. In Figure 6b,c, processed pictures of single DNA spots at the end of the incubation with SiGNPs are shown. Within processed pictures, what is flat on the surface appears as black background, while single particles immobilized on the surface emerge as white dots. From the same pictures, it can be appreciated how streptavidin-functionalized SiGNPs perform better than Utag-functionalized ones (7-fold improvement in the signal). We hypothesize that this divergent performance stems from the necessity for high-affinity interactions to bind bulky objects such as 80 nm AuNPs. For this reason, streptavidin-functionalized SiGNPs are more likely to be immobilized on the surface of positive spots than ssDNA-functionalized ones. To confirm our hypothesis, we performed the same experiment using 40 nm SiGNPs functionalized with both ssDNA and streptavidin. Results, shown in Figure S3, confirm how the signal using ssDNA-functionalized SiGNPs increases by a factor of 3 when using 40 nm nanoparticles instead of 80 nm ones. 

To confirm that our immobilization strategy actually offers advantages in biological assays, we performed a negative control using 80 nm SiGNPs coated with copoly azide 4%, further functionalized with unmodified streptavidin. Lacking DBCO-modification, streptavidin can only adsorb on the surface of nanoparticles. We used these AuNPs with the same experimental procedure (see Supplementary Materials for details). The results underscore two distinct findings: firstly, the binding of SiGNPs on Probe7 spots is considerably lower when using coated SiGNPs where streptavidin is merely adsorbed onto the surface (see Figure S4c,e). This suggests that a lesser amount of streptavidin is immobilized on the surface via adsorption compared to covalent bonding. These findings confirm that copoly azide 4% coating effectively mitigates nonspecific protein interactions with SiGNPs even after prolonged incubation times with high protein concentrations. Secondly, AuNPs with adsorbed streptavidin exhibit a higher degree of adherence to regions of the microarray where they are not intended to bind, thereby augmenting the background signal of the assay (see Figure S4d). This demonstrates how the functionalization outlined in this study can enhance the performance of biological assays by a combination of signal enhancement and noise reduction.

## 4. Discussion

Gold nanoparticles exhibit remarkable versatility in size, ranging from 2 to 250 nm, allowing for precise control over their dimensions. Overall, the stability of gold nanoparticles is essential for ensuring their performance, reliability, and safety across various applications. Stable nanoparticles have a longer shelf life and can be transported over long distances without significant changes in their properties. This is particularly important for commercial applications where consistent product quality is essential. 

Functionalizing gold with biomolecules, especially DNA, represents a promising avenue of research. Thiol-containing small molecules can spontaneously attach to the surface of bulk gold [24]. However, the process of functionalizing gold nanoparticle (AuNP) surfaces with thiol-modified DNA presents challenges due to the poor colloidal stability of AuNPs during conjugation and the negative charges of both citrate-stabilized AuNPs and DNA. Consequently, only a limited number of DNA molecules attach to the AuNPs through simple mixing, and they do so in an uncontrolled manner. This haphazard attachment prevents DNA molecules from effectively hybridizing with complementary DNA (cDNA) due to the strong interactions between DNA bases and AuNPs [25]. Several approaches have been developed to overcome this problem; however, the procedures devised require time-consuming stepwise addition of salt (around 10–50 mM each time), addition of surfactants, sonication, and freezing [26]. Our work aimed at developing a fast, reliable, and scalable coating process capable of stabilizing and functionalizing AuNP in minutes. Various natural and synthetic polymers exhibit the capability to either physically adsorb or covalently bind to gold nanoparticles, depending on their molecular structure and functional groups. The polymers used in this work share a common backbone of poly-dimethylacrylamide (poly-DMA) which carries residues of propyl silane and polyethyleneoxide azide (see Figure 1). The functional group responsible for forming covalent bonds with the silica layer coating the gold surface is the organosilane. We exploit a mechanism that is the combination of physical and chemical adsorption. The formation of a stable coating is facilitated by the propensity of the dimethylacrylamide backbone to adsorb to the silica through a combination of hydrogen bonds and van der Waals forces. We know from previous findings that this class of copolymers forms a thin layer of 2.5 nm on solid surfaces with a density of 1.22 g/cm^3^ when dried [27]. The same is able to swell up to 15 nm in aqueous media [28]. When considering SiGNPs, the poly-DMA backbone of copoly azide 4% initially binds to the silica layer surrounding the nanoparticles by physisorption. The weak interactions between the polymer and the surface are then consolidated by formation of covalent bonds between silanols on the surface and methoxysilane groups on the polymer. The polymer can either envelop the particles or adhere to them in a patchy manner, contingent upon factors such as the particle size, polymer chain length (and structure), and the solvent used. 

Despite copoly azide 4% demonstrated to be rather effective in coating SiGNPs, experimental observations revealed that this polymer fails to effectively stabilize citrate GNPs. A straightforward solution to this issue was identified by incorporating a cationic group into the polymer, obtaining the so-called copoly azide positive 4%. The tertiary amino group of *N*-(3-(dimethylamino)propyl residue pending from the backbone with its opposite charge to that on the particle surface contributes to stabilizing the adsorbed coating. This simple modification to the polymer allows for the production of stable colloidal solutions of polymer-modified GNPs, irrespective of the presence or absence of a silica layer. Usually, to provide a stable coating of GNPs, the duration of incubation may vary depending on factors such as the polymer concentration, nanoparticle size, and temperature, and it can range from a few minutes to several hours. Using copoly azide positive 4%, a remarkably straightforward procedure, involving only one step to ensure uniform coverage and stability of the nanoparticles, was devised.

The polymer modification adds extra features to the AuNPs, whether they are SiGNPs or GNPs. First, by creating a thin layer of moisture around the surface of the nanoparticle, it helps to prevent unwanted substances from sticking to them, thus making the GNPs more stable and less prone to aggregate. Secondly, it facilitates the attachment of bioactive ligands via a reaction involving azide pendants from the polymer backbone.

### 4.1. Characterization of Coated and Functionalized AuNPs 

Uncoated, coated and biofunctionalized nanoparticles were characterized using techniques such as Vis spectroscopy, Nanoparticle Tracking Analysis (NTA) and transmission electron microscopy (TEM) to confirm the successful coating and assess the stability of the nanoparticles.

Vis spectrum provides information on particle size, concentration and aggregation state. The polymer coating does not alter the size of the nanoparticle, its thickness being only in the order of 10 nm [29]. The intensity of the SPR peak, proportional to the concentration of AuNPs in the sample, shows that the recovery after the coating is almost quantitative. Neither the polymer nor the DNA bound to it cause aggregation. No shift to longer wavelengths and broadening of the SPR peak due to plasmonic coupling between adjacent nanoparticles are present in the Vis spectra. The results reported in Figure 2 confirm the stability and lack of interactions of the coated NPs with the surrounding environment.

The successful functionalization was confirmed by Nanoparticle Tracking Analysis (NTA), an advanced technique utilized for the characterization of nanoparticles within diverse solutions. It offers valuable insights into the size distribution and concentration of nanoparticles spanning from approximately 40 to 1000 nm, with the lower detection threshold contingent upon the refractive index of the nanoparticles. In this technique, a laser beam traverses through a suspension containing nanoparticles. As these nanoparticles undergo Brownian motion, they disperse light, and their motions are recorded by a camera. The Brownian motion of nanoparticles correlates with their hydrodynamic radius, and through the analysis of individual particle movements, NTA software processes the captured images, providing information on size distribution and potential presence of aggregates. NTA analysis confirms that the coating does not induce aggregation of the nanoparticles (see Figure 3). 

As a further confirmation, TEM analysis was performed on 40 nm SiGNPs (Figure S1). Comparing the uncoated SiGNPs with the same particles after polymer coating and functionalization with polyT ssDNA, it is evident that the size and the shape of the NPs are not influenced, and the samples do not show any large aggregates (the agglomerate-like effect is due to overlapping of SiGNPs during the drying process), proving that the functionalization does not affect the SiGNPs stability. Moreover, the slightly different aggregation state of the polymer-coated SiGNPs and the ssDNA-functionalized SiGNPs (Figure S1b,c, respectively) suggests that the presence of the oligonucleotide improves the dispersion and the availability of the SiGNPs in solution.

### 4.2. Application of Functionalized AuNPs in Biological Assay

As already mentioned, AuNPs functionalized with biomolecules find wide application in biological assays, especially as labels. For this reason, we demonstrated the success of the SiGNPs functionalization testing their performance in biosensing through an SP-IRIS experiment. In this technology, DNA molecules or streptavidin bound to AuNPs hybridize with complementary oligonucleotides, immobilized on the surface of an SP-IRIS chip. Exploiting functionalized gold nanoparticles for the detection of proteins or DNA presents an exciting opportunity to achieve unparalleled sensitivity and precise quantification, thereby enabling the accurate enumeration of individual particles or molecules. The IRIS (Interferometric Reflectance Imaging Sensor) technology stands out among current state-of-the-art detection systems by providing single-molecule sensitivity through the utilization of simple and cost-effective components: a fundamental sensor substrate, light-emitting diodes (LEDs), an optical setup employing conventional optics, and a CMOS detector. However, the efficacy of nanoparticle labels is paramount to realizing the full potential of this technology. Preventing nonspecific interactions of gold nanoparticles (GNPs) with surfaces other than the target biomolecule can significantly reduce noise and enhance sensitivity. Failure to address this issue may compromise the advantages of the technique, limiting its utility in sensitive detection applications. Therefore, meticulous attention to the quality and specificity of nanoparticle labeling is essential for maximizing the performance and reliability of IRIS technology. The obtained coating enables optimal utilization of the SP-IRIS technology, yielding excellent results in terms of individual nanoparticles detectable within the DNA spot and low levels of counting on the external surface outside the spot and in the negative spot. The coated and functionalized nanoparticles, both with DNA and streptavidin, demonstrate excellent stability in the buffer used during hybridization.

## 5. Conclusions

We developed a functionalization strategy for AuNPs that ensures high stability and fouling-resistant properties, together with enhanced selectivity in biological assays. The use of DMA-based copolymers offers ease of coating and the possibility of further functionalization with both proteins and DNA by using the same reagent. The developed protocol allows great flexibility, which is essential when developing biological assays. In fact, slight changes in copolymer composition allow us to select nanoparticles (in this case, SiGNPs or GNPs) and biomolecules (here represented by, but potentially not limited to, streptavidin and ssDNA) on the basis of assay requirements. The implementation of functionalized AuNPs that ensure high specificity and low background noise, in combination with high-sensitivity detection techniques, surely represents a promising strategy to develop biological assays with unprecedented performance.

## Figures and Tables

**Table 1:** DNA sequences used within this work.

Name	Sequence
Utag-DBCO	5′-CTCAATGTTCGGACTCAG-**DBCO**-3′
Utag-Tag7	5′-**Azide**- CTGAGTCCGAACATTGAGAACAACGATGAGACCGGGCT-3′
Probe2	5′-**Amino**- AAAAAAAAAAAAAAAAAAAATAATCTAATTCTGGTCGCGG-3′
Probe7	5′-**Amino**- AAAAAAAAAAAAAAAAAAAAAGCCCGGTCTCATCGTTGTT-3′
Utag-Biotin	5′-CTCAATGTTCGGACTCAG-**Biotin**-3′
Stabilizer	5′-TTTTTTTTTTTTTTTTTTTT-3′
PolyT-DBCO	5′-**DBCO**-TTTTTTTTTT-3′

## Data Availability

The data that support the findings of this study are available from the corresponding author, D.B., upon reasonable request.

## Supplementary Materials

The following supporting information can be downloaded at: https://www.mdpi.com/article/10.3390/bios14070319/s1, Table S1: NTA data of gold nanoparticle samples; Figure S1: TEM Images of 40 nm SiGNPs: (a) uncoated; (b) coated with copoly azide 4%; (c) functionalized with polyT ssDNA; Figure S2: Relative frequency % distribution of the SiGNP diameters for (a) uncoated SiGNPs, (b) polymer coated SiGNPs, and (c) ssDNA-functionalized SiGNPs; Figure S3: Experiment on SP-IRIS instrument. Bar indicates the signal after 40 min incubation with SiGNPs functionalized with streptavidin and ssDNA; Figure S4: Experiment on SP-IRIS instrument. (a) spotting scheme for silicon chips; (b) Field of view (FOV) after incubation with copoly azide 4% coated 80 nm SiGNPs functionalized with DBCO-modified streptavidin; (c) enlargement of a spot of Probe7 after incubation with copoly azide 4% coated 80 nm SiGNPs functionalized with DBCO-modified streptavidin; (d) FOV after incuba-tion with copoly azide 4% coated 80 nm SiGNPs functionalized with adsorbed streptavidin; (e) enlargement of a spot of Prove7 after incubation with copoly azide 4% coated 80 nm SiGNPs func-tionalized with adsorbed streptavidin.
