# Supplementary material for: Copolymer-Coated Gold Nanoparticles: Enhanced Stability and Customizable Functionalization for Biological Assays
Source: Biosensors (Basel). 2024 Jun 24;14(7):319. doi: 10.3390/bios14070319 (PMC11274550; doi:10.3390/bios14070319)
Supplement: Supplementary file 1 [file biosensors-14-00319-s001.zip › biosensors-3034494-supplementary.pdf]

biosensors

MDPI

Supplementary Material

Article

Copolymer-Coated Gold Nanoparticles: Enhanced Stability and Customizable Functionalization for Biological Assays

Dario Brambilla [1,*], Federica Panico [1], Lorenzo Zarini [1], Alessandro Mussida [1], Anna M. Ferretti [2], Mete Aslan [3], M. Selim Ünlü [3] and Marcella Chiari [1]

[1] Institute of Chemical and Technological Science "Giulio Natta", National Research Council of Italy, via privata Mario Bianco 9, 20131 Milan, Italy

[2] Institute of Chemical and Technological Science "Giulio Natta", National Research Council of Italy, via Gaudenzio Fantoli 16/15, 20138 Milan, Italy

[3] Electrical and Computer Engineering Department, Boston University, Boston, MA 02215, USA

* Correspondence: dario.brambilla@scitec.cnr.it

Table S1. NTA data of gold nanoparticle samples.

Name	Dilution factor	Mean size (nm)	Mode size (nm)	Concentration (particles/ml)
Uncoated GNPs 40nm	1:1000	66.3 +/- 1.1	45.7 +/- 0.5	3.57e+08 +/- 8.63e+06
Coated GNPs 40nm	1:500	72.7 +/- 0.7	63.9 +/- 1.6	5.03e+08 +/- 1.11e+07
Streptavidin functionalized GNPs 40nm	1:500	68.1 +/- 1.2	54.8 +/- 0.7	3.24e+08 +/- 9.43e+06
Uncoated SiGNPs 40 nm	1:1000	101.6 +/- 4.7	82.1 +/- 6.4	7.28e+07 +/- 3.78e+06
Coated SiGNPs 40 nm	1:500	106.4 +/- 1.5	81.5 +/- 5.2	1.35e+08 +/- 3.26e+06
ssDNA functionalized SiGNPs 40 nm	1:1000	103.3 +/- 3.2	67.5 +/- 2.2	6.76e+07 +/- 5.01e+06
Streptavidin functionalized SiGNPs 40 nm	1:1000	93.5 +/- 2.0	72.4 +/- 2.1	7.72e+07 +/- 2.69e+06
Uncoated SiGNPs 80 nm	1:1000	90.7 +/- 0.5	97.9 +/- 1.9	1.69e+08 +/- 5.95e+06
Coated SiGNPs 80 nm	1:1000	123.1 +/- 4.0	114.6 +/- 1.4	3.23e+07 +/- 8.99e+05
ssDNA functionalized SiGNPs 80 nm	1:1000	107.2 +/- 0.6	104.7 +/- 2.1	2.40e+08 +/- 5.39e+06
Streptavidin functionalized SiGNPs 80 nm	1:1000	154.1 +/- 4.6	109.5 +/- 3.7	5.62e+07 +/- 4.10e+06

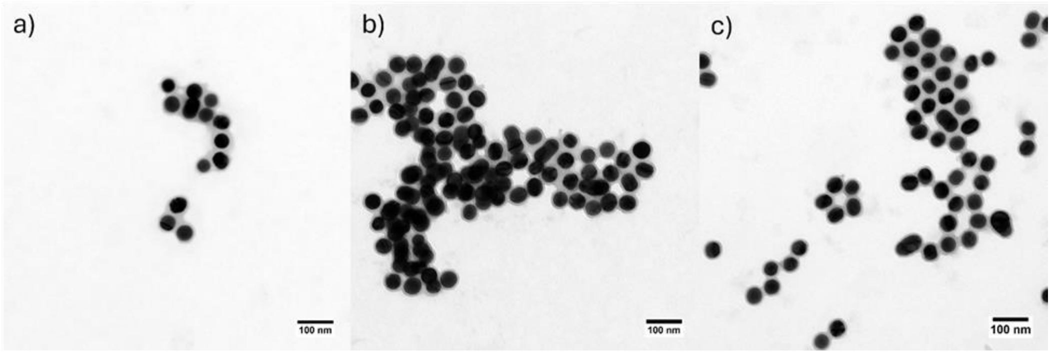

Figure S1: *TEM Images of 40 nm SiGNPs: (**a**) uncoated; (**b**) coated with copoly azide 4%; (**c**) functionalized with polyT ssDNA.*

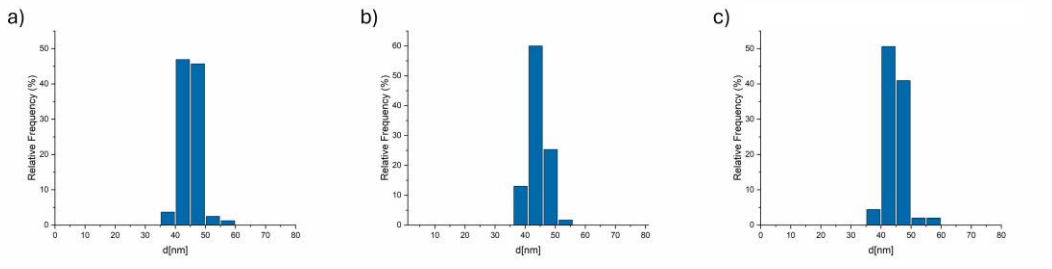

Figure S2. *Relative frequency % distribution of the SiGNP diameters for (**a**) uncoated SiGNPs, (**b**) polymer coated SiGNPs, and (**c**) ssDNA-functionalized SiGNPs.*

S.1 Functional Test using ssDNA-functionalized SiGNPs

SP-IRIS chips were functionalized with Probe2 and Probe7 (the negative and the positive spots, respectively) as described in Section 2.5. Chips were mounted on the support slide and positioned inside the SP-IRIS instrument. Chips were washed by flushing twice 500 µL of MQ water and once 500 µL of 2X SSC (each washing step was performed at 500 µL/min). Chips were incubated with a solution of 10 nM Utag-Tag7 in 2X SSC for 40 minutes at 10 µL/min. At the end of the first incubation, chips were washed by flushing 2X SSC and then incubated with 0.1 OD of Utag-functionalized SiGNPs (prepared as described in Section 2.10) in 2X SSC for 40 minutes at 10 µL/min. During the incubation, a single image was acquired every 2 minutes. Then, acquired images were processed and SiGNPs immobilized on spots counted using ImageJ software.

S.2 Functional test using streptavidin-functionalized SiGNPs

SP-IRIS chips were functionalized with Probe2 and Probe7 (the negative and the positive spots, respectively) as described in Section 2.5. Chips were mounted on the support slide and positioned inside the SP-IRIS instrument. The chip was washed by flushing twice 500 μL of MQ water and once 500 μL of 2X SSC (each washing step was performed at 500 μL/min). The chip was incubated with 10 nM Utag-Tag7 in 2X SSC for 40 minutes at 10 μL/min. At the end of the incubation, the chip was washed by flushing 2X SSC and then incubated with 100 nM Utag-Biotin in 2X SSC for 40 minutes at 10uL/min. At the end of the incubation, the chip was washed by flushing 2X SSC and then incubated with 0.07 OD solution of streptavidin-functionalized SiGNPs in 2X SSC for 40 minutes at 10 μL/min. During the last incubation, a single image was acquired every 2 minutes. Then, acquired images were processed and particles immobilized on spots counted using ImageJ software.

S.3 Comparison of 40 nm and 80 nm SiGNPs for biological test

In order to confirm our hypothesis that low signals obtained using 80 nm SiGNPs functionalized with ssDNA (see Figure 6 in the main text) could be attributed to unsufficient strenght offered by DNA-DNA interactions to graft and immobilized bulky objects like 80 nm nanoparticles, we repeated the same experiment using 40 nm SiGNPs functionalized with either streptavidin and ssDNA. It is appropriate to preface that SP-IRIS instrument is optimized for the detection of 80 nm AuNPs, and that some of the 40 nm AuNPs used within this experiment may go undetected.

Results are shown in Figure S3. As expected, the signal using streptavidin-functionalized SiGNPs decreases using 40 nm particles due to lower sensitivity from the instrument. Nevertheless, in the case of ssDNA-functionalized SiGNPs, shifting to 40 nm particles produced a 3-fold enhancement of the signal. This results confirm that DNA-DNA interaction is not sufficient to provide an effective immobilization of 80 nm AuNPs, while maintaining its efficacy with smaller nanoparticles. To be noted, streptavidin-functionalized SiGNPs provides higher signals in comparison with ssDNA-decorated SiGNPs using both sizes of nanoparticles.

This is particularly important since it highlights how – within the same biological assay – the size of AuNPs should be optimized on the basis of their functionalization strategy. This reinforces once again the importance of the functionalization strategy proposed within this work, given the flexibility offered by our approach.

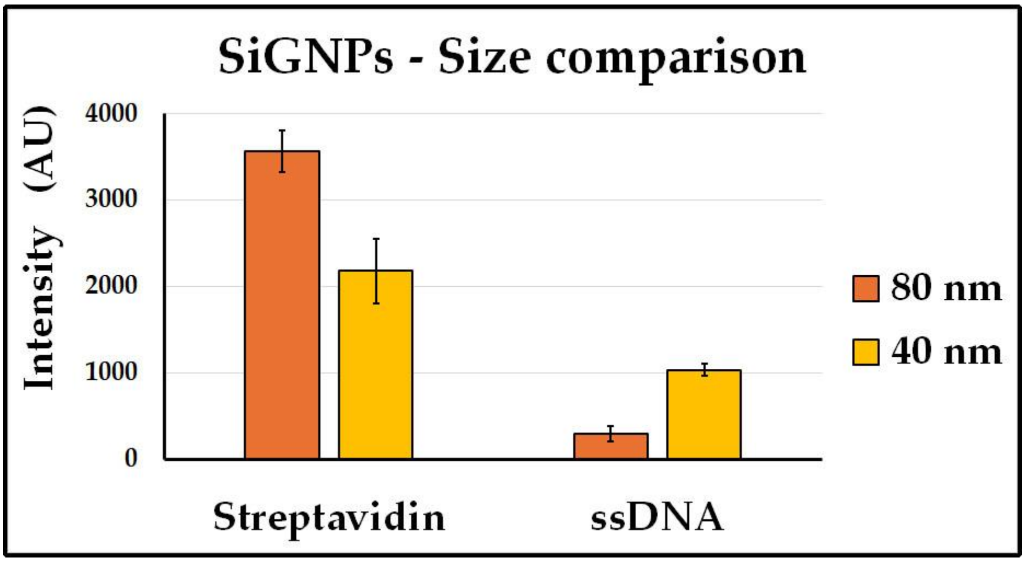

Figure S3. *Experiment on SP-IRIS instrument. Bar indicates the signal after 40 min incubation with SiGNPs functionalized with streptavidin and ssDNA.*

S.4 Adsorption of streptavidin on copoly azide 4% coated SiGNPs

To a 200 µl solution of 80 nm SiGNPs coated with copoly azide 4% (prepared as described in Section 2.7) 30 µl of 2 mg/ml unmodified streptavidin, 70 µl of MQ water and 0.2 µl of Tween 20 were added and the obtained solution was incubated overnight at 25°C under stirring. After the incubation, the sample was centrifuged for 5 minutes at 12,000 x g, the supernatant was removed, and SiGNPs resuspended in 300 µL of 0.1X PBS + 0.05% Tween 20. Centrifugation was repeated three times to wash SiGNPs. Finally, SiGNPs were redispersed using an immersion sonicator.

S.5 Functional test using streptavidin-adsorbed onto SiGNPs

SP-IRIS chips were functionalized with Probe2 and Probe7 (the negative and the positive spots, respectively) as described in Section 2.4 in the main text. Chips were mounted on the support slide and positioned inside the SP-IRIS instrument. The chip was washed by flushing twice 500 µL of MQ water and once 500 µL of 2X SSC (each washing step was performed at 500 µL/min). The chip was incubated with 10 nM Utag-Tag7 in 2X SSC for 40 minutes at 10 µL/min. At the end of the incubation, the chip was washed by flushing 2X SSC and then incubated with 100 nM Utag-Biotin in 2X SSC for 40 minutes at 10uL/min. At the end of the incubation, the chip was washed by flushing 2X SSC and then incubated with 0.07 OD solution of streptavidin-adsorbed SiGNPs (prepared as described in Section S.1) in 2X SSC for 40 minutes at 10 µL/min. During the last incubation, a single image was acquired every 2 minutes. Then, acquired images were processed and particles immobilized on spots counted using ImageJ software.

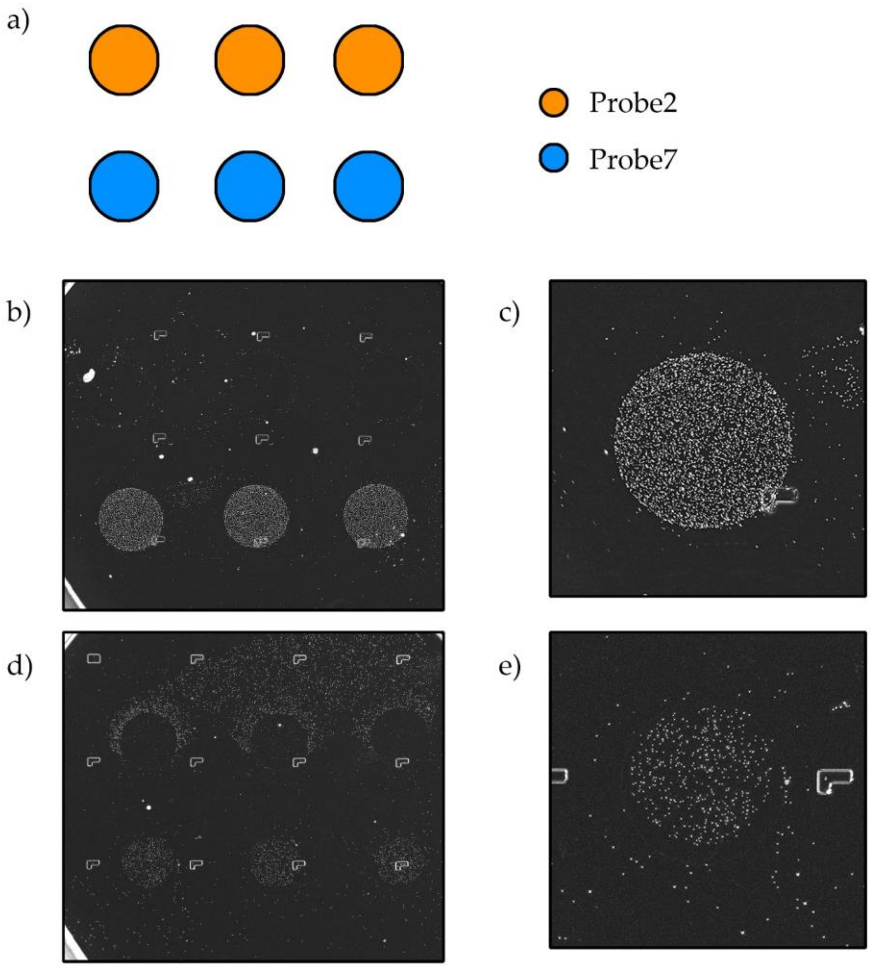

Figure S4. *Experiment on SP-IRIS instrument. (**a**) spotting scheme for silicon chips; (**b**) Field of view (FOV) after incubation with copoly azide 4% coated 80 nm SiGNPs functionalized with DBCO-modified streptavidin; (**c**) enlargement of a spot of Probe7 after incubation with copoly azide 4% coated 80 nm SiGNPs functionalized with DBCO-modified streptavidin; (**d**) FOV after incubation with copoly azide 4% coated 80 nm SiGNPs functionalized with adsorbed streptavidin; (**e**) enlargement of a spot of Prove7 after incubation with copoly azide 4% coated 80 nm SiGNPs functionalized with adsorbed streptavidin.*

In Figure S4a the spotting scheme used for the functionalization of silicon chips is depicted. The same spotting scheme can be found in Figures S4b and S4d. After images are processed, the chip appears as black regions, where white dots represent single SiGNPs bound to the surface.

As it can be seen from Figure S4b, when using SiGNPs functionalized with covalently-bound streptavidin a strong signal is produced on Probe7 spots (see enlargement of a single spot in Figure S4c) while only few particles sticked both onto negative control (i.e. Probe2 spots) and the background. On the contrary, when streptavidin is merely adsorbed onto SiGNPs, lower signals are detected on Probe7 spots (see Figure S4e for the enlargement of a single spot). Additionally, despite no signal is detected on negative spots (Probe2 spots) a larger amount of SiGNPs is nonspecifically captured by the background, especially in the area surrounding negative control spots.

Considering this results both in terms of signal intensity and background noise, it clearly appears how the covalent immobilization of streptavidin on SiGNPs represents a better strategy to devise a biological assay.
